# Factors Associated with the COVID-19 Vaccination Status of Higher Education Students: Results of an Online Cross-Sectional Survey at Six Universities in Southwestern Germany

**DOI:** 10.3390/vaccines10091433

**Published:** 2022-08-30

**Authors:** Anna T. Neunhöffer, Jolanda Gibilaro, Anke Wagner, Jana Soeder, Benjamin Rebholz, Gunnar Blumenstock, Peter Martus, Monika A. Rieger, Esther Rind

**Affiliations:** 1 Institute of Occupational and Social Medicine and Health Services Research, University Hospital Tübingen, Wilhelmstr. 27, 72074 Tübingen, Germany; 2 Institute for Clinical Epidemiology and Applied Biometry, University Hospital Tübingen, Silcherstr. 5, 72076 Tübingen, Germany

**Keywords:** COVID-19 pandemic, vaccination acceptance, university setting, infection control, health and safety measures, occupational health services research, cross-sectional survey

## Abstract

This cross-sectional study explored factors associated with the corona virus disease 2019 (COVID-19) vaccination acceptance among higher education students in southwestern Germany. We conducted a cross-sectional online survey at six state-approved higher education institutions (HEIs) between July and November 2021. In addition to descriptive analyses, univariate as well as multivariate binary logistic regression analyses were conducted. A total of 6556 higher education students aged 18 years and older participated in our survey; 91.4% of participating students had been vaccinated against COVID-19 at least once. The factors that significantly contributed to the explanation of higher education students’ vaccination status in the multivariate analysis (area under curve—AUC = 0.94) were variables on the perception of the virus SARS-CoV-2 (affective risk perception: Adjusted odds ratio—aOR = 1.2; perception of the outbreak as a media-hype: aOR = 0.8), attitudes towards personal (aOR = 0.7) and study-related (aOR = 0.8) health and safety measures to prevent transmission of SARS-CoV-2, and attitudes towards COVID-19 vaccination (preservation of own health: aOR = 1.3; confidence in vaccine safety: aOR = 1.7; supporting higher education through vaccination: aOR = 1.2; own contribution to the containment of the pandemic: aOR = 1.7). The findings target assisting HEIs in returning to face-to-face teaching after previous semesters of online teaching.

## 1. Introduction

Since March 2020, the World Health Organization (WHO) has declared the coronavirus disease 2019 (COVID-19) caused by SARS-CoV-2 (Severe Acute Respiratory Syndrome Coronavirus 2) a pandemic [1]. Consequently, measures to reduce the transmission of SARS-CoV-2 were introduced covering various areas of daily life. The most prominent generally recommended and in the further course of the pandemic partly mandatory rules comprised social distancing, personal hygiene (especially when washing hands, coughing and sneezing), wearing mouth-nose protection and regular SARS-CoV-2 antigen testing [2]. These measures to protect people against transmissions of SARS-CoV-2 are behavioral preventive measures. In addition, with the approval of COVID-19 vaccines at the end of 2020, vaccination has become one of the most effective behavioral preventive measures to prevent COVID-19 [3], reducing infection rates, hospitalizations and mortality [3]. Before winter 2021, four vaccines received conditional marketing approval in the European Union (EU) by the European Medicines Agency (EMA), including two mRNA (messenger RNA) vaccines [4]. Various activities have already been developed and implemented in the EU countries to increase the acceptance and uptake of COVID-19 vaccination in the population [5].

### 1.1. Vaccine Hesitancy

Because COVID-19 vaccines have been rapidly developed and given conditional marketing approval, the factors influencing vaccination acceptance were and are highly relevant. Already in 2019, the WHO has highlighted general vaccine hesitancy as one of ten threats for global health [6]. Vaccine hesitancy can be defined as “the delay in acceptance or refusal of vaccination despite availability of vaccination services” [7]. However, the term has been debated in the literature, and studies on COVID-19 vaccination have used a variety of measures to describe and predict “vaccine hesitancy” [8,9,10,11] including, for example, outcomes such as “vaccination readiness”, “vaccine uptake” (see for example [12,13]) or “vaccination acceptance” [14,15]. According to the definition of the Strategic Advisory Group of Experts on Immunization (SAGE) Working Group on Vaccine Hesitancy, hesitancy has been described as a continuum between complete acceptance and complete rejection of available vaccines [7].

Moreover, vaccination acceptance can change over time [16,17]. Therefore, it is important to consider the epidemiological, socioeconomic and cultural context in which COVID-19 vaccination studies take place. For example, a considerable amount of research has shown that there are differences regarding the acceptability of COVID-19 vaccination in different cultures and populations [18]. Consequently, many studies have been conducted in general populations in countries around the world (see for example [16,19,20,21,22]) as well as in special populations such as healthcare students and professionals (see for example [23,24,25,26,27,28,29,30]). Furthermore, individual factors including age, gender and educational level have been broadly examined with COVID-19 vaccination acceptance [22].

### 1.2. Higher Education Students

One particular group of people who have been seriously affected by the pandemic are higher education students: About 220 million higher education students worldwide experienced a complete disruption of their daily study routine [31]. In Germany, as well as in other parts of the world, higher education institutions (HEIs) were obliged to implement structural preventive measures, including the discontinuation of face-to-face teaching, the closing of facilities on campus and transitioning employees and students to work and learn from home [32,33]. State regulations required students to suddenly switch to online teaching. This transition to protect against infections with SARS-CoV-2 and ensure a safe working and learning environment required higher education students to adapt in numerous ways. This also had an effect on physical and mental health. Higher education students have been even more negatively affected by the impact of the COVID-19 pandemic compared to the general population [34]. Knight et al. [35], for example, describe the detrimental impact of the COVID-19 pandemic on students and staff in higher education in their qualitative study. During the COVID-19 pandemic, a decrease in subjective wellbeing [36,37] and an increase in negative emotional symptoms was reported among higher education students [38]. Uncertainty about the conducting of courses and exams increased stress for students, leading to an intensification of negative emotional symptoms [38] and future career anxiety [39]. Further drastic changes faced by higher education students in the COVID-19 pandemic could include loneliness, financial difficulties, deterioration in health behaviors, increasing mental health issues [40], personal adjustments in light of changing study conditions and online teaching and dealing with technical malfunctions during digital teaching [41]. During the COVID-19 pandemic, teaching and thus the training of a large part of the future workforce has been challenging. To minimize the negative impact, adherence to protective and hygienic measures has been essential so that face-to-face classes can continue as the pandemic progresses.

The proportion of face-to-face teaching has varied between HEIs during the COVID-19 pandemic and also depends on teaching subject cultures and the available space at the HEIs [42]. In Germany, 1 April 2020 marked the beginning of the period in which the physical campuses of HEIs were completely closed and only online teaching was offered [33]. Face-to-face teaching was not reintroduced as a standard practice at universities in the state of Baden-Württemberg (BW) in southwestern Germany until the COVID-19 regular study procedure from September 2021 [43]. The regulation only permitted students the participation in indoor classes with proof of vaccination, recovery from a COVID-19 infection or a current negative rapid antigen test result; separate protective measures for persons belonging to a risk group for severe COVID-19 courses [44] were not part of this regulation anymore [43]. In Germany, anyone who wanted to be vaccinated against COVID-19 could accept a vaccination offer in the summer of 2021 [45]. Vaccination acceptance as well as the acceptance of other preventive measures among higher education students has significant implications for further planning of teaching in HEIs, as well as for the modifications of protective and hygienic measures within the context of higher education teaching. Hence, it is important to study students’ attitudes towards infection control measures, and we expect our research to provide new evidence on factors associated with COVID-19 within the higher education system.

### 1.3. Research Questions

Within this context, the following research questions will be considered:

(1) How do students in higher education evaluate behavioral and structural measures to prevent SARS-CoV-2 infections in the study environment?
(2) Which factors are associated with the COVID-19 vaccination status of higher education students when they had all been offered vaccination?

## 2. Materials and Methods

This cross-sectional study is part of a comprehensive exploratory mixed-methods project investigating pandemic management across different companies and workplaces in Germany between August 2020 and November 2021 [46]. Over the study period, we also conducted standardized employee surveys in different companies and workplaces to explore attitudes toward health and safety measures implemented to prevent SARS-CoV-2 infections in the working environment [46]. Furthermore, the Ministry of Science, Research and the Arts (“Ministerium für Wissenschaft, Forschung und Kunst”) Baden-Württemberg requested HEIs in fall 2021 to provide information on students’ COVID-19 vaccination status in order to better plan the COVID-19 study procedure [43] including behavioral and structural prevention for studying at HEIs over the winter semester 2021/2022 [47]. Therefore, we extended the original employee survey to include students [47].

This article refers to the online survey of students at six state-approved HEIs in the federal state BW in southwestern Germany. We investigate variables likely to be associated with the COVID-19 vaccination status of higher education students before or at the restart of face-to-face teaching. We chose an exploratory analysis approach since there has been little evidence of plausible behavioral and structural correlates of higher education students’ vaccination status [48].

### 2.1. Study Setting

The surveys were conducted after previous semesters of online teaching [42]. The time frame of our cross-sectional survey of students studying at various HEIs in the state BW covers July to the beginning of November 2021. This period describes the time before or the very beginning of a new semester with face-to-face teaching under specified protective and hygienic regulations, as the start of the winter semester varied across HEIs [43]. During the start of the survey period, COVID-19 incidences were at a relatively low level: the 7-day incidences per 100,000 inhabitants in BW at the end of July were at 10, with 7-day incidences per 100,000 inhabitants rising to 225 at the end of the surveyed period [49]. The assumption made by the Federal Ministry of Health at the beginning of the survey period was that the more contagious delta variant of SARS-CoV-2 would dominate the occurrence of infections in Germany in fall and winter 2021 [45].

### 2.2. Study Population

The study population comprised enrolled students with a minimum age of 18 years. The higher education students participated voluntarily in the survey on the topic of “COVID-19 vaccination” and consented to the anonymous processing of their data. In the winter semester 2021/2022, about 39,300 students were registered at the six participating HEIs in BW. Higher education students from all programs of study and degrees were eligible to take part in the survey.

### 2.3. Recruitment and Data Collection

All HEIs in BW were required to develop a hygiene concept for face-to-face teaching on the basis of the students’ COVID-19 vaccination status. Therefore, all students of the six HEIs were invited to take part in the online survey by the representatives of the HEIs via the institutions mailing list. In the middle of the survey period, a one-time reminder was sent to all students to take part in the survey. Participation in the survey required about 15 min. The survey language was German and we used the survey tool Unipark [50].

### 2.4. Development of the Standardized Online Student Survey

In our survey, higher education students were asked next to their COVID-19 vaccination status whether and how they perceive or have perceived existing vaccination offers and how they assess and evaluate preventive measures against the spread of SARS-CoV-2 in the study environment. The questionnaire was designed by an interdisciplinary team of health scientists, health services researchers and practitioners in occupational medicine. The questions were part of previous employee surveys [46]. For this reason, the questionnaire was pretested among employees of various companies. The original employee survey was adapted in agreement with university representatives. We modified the approach to students, including, for example, faculties and programs of study at all participating HEIs.

### 2.5. Elements of the Standardized Online Student Survey

The survey instrument was a standardized questionnaire; all participants were asked about these five topics:

- **Individual variables (socio-demographic and study-related characteristics)**
  a. Age (numerical variable); categorical variables: gender, affiliation to risk group for developing severe COVID-19 courses [44], SARS-CoV-2 infection, COVID-19 vaccination status, German or other nationality, part-time job while studying, living in a committed relationship, number of household members, presence of health professional within household
  b. Big Five personality trait (BFI-10 [51]—five subscales consisting of two items with the range 1–5—low to high each)
  c. Social Desirability–Gamma Short Scale (KSE-G [52]—two subscales consisting of three items with the range 1–5—low to high each) [53]
  d. Affiliation to and type of HEI (study-related categorical variables: five dummy-coded variables and application-oriented/research-oriented)
  e. Faculty/program of study (study-related categorical variable: healthcare university curriculum/non-healthcare university curriculum)
- **Perception of SARS-CoV-2 in general** [54]
  a. Disease perception (scale consisting of two items with the range 1–7—low to high disease perception)
  b. Affective risk perception (scale consisting of three items (e.g., worry or thinking about the coronavirus all the time [55]) with the range 1–7—low to high affective risk perception)
  c. Perceived adequacy of media coverage (7-point Likert scale with the range from ‘too little media attention’ to ‘media-hype’)
  d. Perceived personal susceptibility (7-point Likert scale with the range from ‘not susceptible at all’ to ‘very susceptible’)
  e. Expected severity of COVID-19 disease for one’s own (7-point Likert scale with the range from ‘totally harmless’ to ‘extremely dangerous’)
- **Attitude toward health and safety measures to prevent SARS-CoV-2 infections in the study environment**
  a. Attitude toward behavioral preventive measures in the study environment (score consisting of eight items measured on a 5-point Likert scale ranging from 1 ‘not at all suitable’ to 5 ‘very suitable’)
  b. Attitude toward structural preventive measures in the study environment (scale consisting of nine items measured on a 5-point Likert scale ranging from 1 ‘not at all suitable’ to 5 ‘very suitable’)
- **Impact of COVID-19 on the personal environment**
  a. Perceived probability to contract COVID-19 in private surroundings (7-point Likert scale with range low to high)
  b. Perceived probability to contract COVID-19 in current campus surrounding (7-point Likert scale with range low to high)
  c. Readiness to perform SARS-CoV-2 rapid antigen tests (7-point Likert scale with range ‘in no case’ to ‘in any case’)
  d. Confirmed SARS-CoV-2 infection (categorical variable: no confirmed infection/confirmed infection)
  e. COVID-19 specific reactance (score consisting of four items with range 1–7 low to high reactance) [54]
  f. COVID-19 specific resilience (score consisting of four items with range 1–7 low to high resilience) [54]
  g. Trust in fellow students to adhere to distance and hygiene rules (7-point Likert scale, each with a range from low to high adherence)
- **Variables relating to COVID-19 vaccination**
  a. Attitude toward COVID-19 vaccination (own health, avoidance of personal disadvantages of the pandemic [54], avoidance of disadvantages of the pandemic for HEIs on 7-point Likert scale with the range ‘do not agree at all’ to ‘fully agree’)
  b. COVID-19 vaccination status (categorical variable: not yet vaccinated/vaccinated at least once against COVID-19)
  c. 5C psychological antecedents of vaccination (confidence, complacency, constraints, calculation and collective responsibility) [56] in relation to COVID-19 vaccination (five items measured on 7-point Likert scale with range ‘do not agree at all’ to ‘fully agree’)
  d. Personal assessment of, among others, the benefits and risks of COVID-19 vaccination (eight items measured on a 7-point Likert scale with the range from ‘do not agree at all’ to ‘fully agree’) [54,57].

### 2.6. Statistical Analysis

All analyses were performed with IBM Statistics SPSS for Windows, version 28 (IBM Corp., Armonk, NY, USA). Numerical variables (age, scores) were described with mean, standard deviations, median and range, while categorical variables were described with frequencies and percentages including and excluding missing values. The scores describing the attitudes toward health and safety measures to prevent SARS-CoV-2 infections were computed with mean across available items [58]. We address differences between higher education students from different institutions by controlling for their affiliation in our multivariate analyses. We focus on factors that are likely to be associated with vaccination status (0—not yet vaccinated/1—vaccinated at least once against COVID-19) of higher education students using binary logistic regression analysis. Our thematic groups of explanatory variables are (I) individual variables (socio-demographic and study-related characteristics), (II) perception of SARS-CoV-2 in general, (III) attitude toward health and safety measures to prevent SARS-CoV-2 infections, (IV) impact of COVID-19 on the personal environment and (V) variables relating to COVID-19 vaccination.

The possible explanatory variables of students’ COVID-19 vaccination status were related to the dependent variable (0/1) either as metric or categorical variables with dummy coding (0—no/1—yes). To select possible explanatory variables/factors for the multivariate logistic regression model, the regression coefficients of univariate logistic regressions were considered initially (see Table S1, Supplementary Materials). In the multivariate binary logistic regression model, variables that were theoretically justifiable and had shown a significant univariate association (*p* < 0.05) with the outcome variable “COVID-19 vaccination status” were tested in the variable groups described earlier.

All variables with a significant influence on the outcome variable of each group from the questionnaire were checked for collinearity (*r* < 0.7); those variables that made a higher contribution to explaining the outcome were chosen. The selected variables were used to calculate the final model using the “enter” method [59]. In steps, these single possible explanatory variables were included in the multivariate binary logistic regression model in different blocks (=variable group from the questionnaire) until a model emerged that explained the COVID-19 vaccination status of the students as well as possible and was theoretically plausible. The explanatory variables were grouped thematically (left column of Table S1, Tables 2 and 3). First, the control variables (affiliation with the HEIs and social desirability [52]) were entered. Variables belonging to the three thematic groups (II) “Perception of SARS-CoV-2 in general”, (III) “Attitude toward health and safety measures to prevent SARS-CoV-2 infections”, and (V) “Variables relating to COVID-19 vaccination” remained with significant influence on students’ COVID-19 vaccination status in the multivariate binary logistic regression model.

We show the model fit using the Hosmer and Lemeshow test [59] as each block of variables is added (Table 2, Results). Regression coefficients (B), Wald statistics, *p*-values, and adjusted odds ratios (aOR) with their respective two-sided 95% confidence intervals are reported for the final multivariate binary logistic regression model (Table 3, Results). The n = 177 cases (2.7%) with values missing at random were not imputed and not included in the multivariate binary logistic regression analysis; listwise valid cases were included in the multivariate binary logistic regression analysis. Outliers with studentized residuals ±3 in the multiple logistic regression analysis were observed—a sensitivity analysis was performed to compare results with and without outliers. If the identified outliers do not meaningfully affect the model fit of the multivariate binary logistic regression model, we reported the results of the model with outliers. The multivariate binary logistic regression model was controlled for affiliation with the HEIs surveyed and response behavior by social desirability [52] (aOR in Table 3, Results). Receiver operating characteristics (ROC) analysis was performed to quantify the prediction of the final multivariate model [60]. We report the area under the curve (AUC) including a test of significance vs. hypothesis H0: AUC = 0.5 (random chance).

### 2.7. Ethical Considerations

The study was approved by the responsible local ethical committee of the Medical Faculty, University of Tübingen and University Hospital Tübingen (No. 423/2020BO). Only study participants who agreed to anonymous analyses of their data and completed the survey in full were included in the analyses; participants were free to quit the survey at any time.

## 3. Results

### 3.1. Characteristics of the Participants

A total of 6556 higher education students from six state-approved HEIs participated in our online survey on COVID-19 vaccination. A total of 13.6% of students (n = 893) from four application-oriented HEIs and 86.4% of students (n = 5663) from two research-oriented HEIs participated. The overall response rate was about 6%; the response rates per HEI ranged from 5 to 32%. Participation per HEI ranged from 0.3% (n = 21) to 61.6% (n = 4036). About 11% (n = 694) of the participating higher education students reported attending a degree program in health care. Students in the non-healthcare programs of study were the majority (valid percentage: 89.3%; n = 5769).

The mean age of the respondents was 24 years (SD = 4.2; range: 18–70 years). The majority of participants were female (64.8%; n = 4106), and 91.4% (n = 5935) had at least received one dose of the COVID-19 vaccine. Reasons against COVID-19 vaccination provided by unvaccinated participating higher education students in free texts included, for example, insufficient research on vaccines, the conditional market approval of the vaccines, low risk perception of COVID-19 in general, and considering oneself to be young and in very good health. Socio-demographic and personal characteristics of all respondents are shown in Table 1.

### 3.2. Attitude toward Health and Safety Measures to Prevent SARS-CoV-2 Infections (Research Question 1)

The attitudes toward health and safety measures to prevent SARS-CoV-2 infections were very positive (scale from 1 ‘extremely negative’ to 5 ‘extremely positive’). We asked the higher education students about the appropriateness of personal protective and hygienic measures such as maintaining a safety distance of 1.5 m from other people, adhering to proper coughing and sneezing behavior, and staying at home in case of symptoms of illness. Overall, the participating higher education students rated the appropriateness of behavioral preventive measures to prevent SARS-CoV-2 infections with a mean of 4.28 (SD = 0.56; n = 6526).

Examples of study-related protective and hygienic measures were to form fixed study groups, to avoid unnecessary contacts in high-traffic areas, or to clean lecture halls on a regular basis. These study-related, structural preventive measures were rated by all higher education students surveyed on a scale from 1 ‘extremely negative’ to 5 ‘extremely positive’ with a mean of 3.75 (SD = 0.71; n = 6521).

### 3.3. Univariate Binary Logistic Regression Analysis (Preparation for Answering Research Question 2)

Individual and study-related variables were put univariately in relation to the COVID-19 vaccination status of the participating higher education students. We report these univariate binary logistic regression results for each thematic variable group; we also use these thematic blocks to subsequently build the multivariate binary logistic regression model. Table S1 in the Supplementary Materials presents the univariate binary logistic regression analyses for individual variables and other variable groups from the questionnaire.

When examining individual variables (variable group I, not directly related to COVID-19), German nationality, part-time job while studying, and studying in medicine or health had a significant positive association on the vaccination status and acceptance of COVID-19 vaccination among all participating higher education students. If the participant had a health professional in their household and was in a committed relationship, or if the personality traits conscientiousness and openness to experiences were stronger pronounced, this was negatively correlated with being vaccinated against COVID-19.

All variables related to the perception of SARS-CoV-2 in general (variable group II) were significantly associated with COVID-19 vaccination status. For example, the more severe an infection or the more susceptible the participating individual perceived oneself at risk for a SARS-CoV-2 infection, the more likely the individual had already received a COVID-19 vaccination. However, if respondents considered the outbreak of SARS-CoV-2 as a media hype, they were less likely to report a COVID-19 vaccination.

Univariate, both attitudes toward the appropriateness of personal and study-related health and safety measures (variable group III) had a significant positive association with the participants’ COVID-19 vaccination status. Variables related to the impact of SARS-CoV-2 and the COVID-19 pandemic on the personal environment (variable group IV) affected higher education students’ COVID-19 vaccination status to varying degrees. In contrast, all variables directly related to COVID-19 vaccination were significantly related to their COVID-19 vaccination status (variable group V). The strongest effects on the COVID-19 vaccination status were the two variables on confidence in the safety of COVID-19 vaccination and the positive intention to contribute positively to the mitigation of the pandemic through vaccination.

### 3.4. Multivariate Binary Logistic Regression Analysis (Research Question 2)

Table 2 shows the model summaries with the addition of each variable block and the area under the ROC curve (AUC) with a maximum value of “1” (perfect accuracy).

The Hosmer and Lemeshow test with the last block for the multivariate model was non-significant (χ^2^(8) = 5.424; *p* = 0.711). The multivariate binary logistic regression model was a significant improvement in fit over the null model (χ^2^(24) = 166.47; *p* < 0.001) and explained 61.0% of the total variance (Nagelkerke Pseudo R^2^ = 0.61) of the higher education students COVID-19 vaccination status. By adding the block “Attitude toward health and safety measures to prevent SARS-CoV-2 infections”, AUC improved only slightly from 0.831 to 0.833.

The multivariate binary logistic regression model, which included n = 6356 cases excluding 20 identified outliers, yielded in χ^2^ = 2105.90, df = 15, *p* < 0.001 and explained 65.3% of the total variance (Nagelkerkes Pseudo R^2^ = 0.653).

Table 3 shows the results for the estimation of students’ COVID-19 vaccination status (n = 6376, including the 20 identified outliers) with our selected explanatory variables.

A total of eight factors form the multivariate binary logistic regression model: two variables of the group “Attitude toward health and safety measures to prevent SARS-CoV-2 infections”, two variables of the group “Perception of SARS-CoV-2 in general” and four variables relating to COVID-19 vaccination contributed significantly to predicting the COVID-19 vaccination status of higher education students. The effect of the attitude toward health and safety measures is reversed as soon as the variables relating to COVID-19 vaccination are included. The attitudes toward health and safety measures are significantly correlated in the multiple binary logistic regression (*r* = −0.571); the correlation between the attitudes and the vaccine-related variables is close to zero. Correlations outside the multivariate model between the attitudes correlate highly significantly at *r* = 0.645, providing evidence that the included explanatory variables of higher education students’ COVID-19 vaccination status are mediators rather than predictors.

In the first block of the multivariate binary logistic regression model, the five dummy-coded variables for affiliation with HEIs and the two social desirability response scores were included to control the ORs hereunder (aOR).

If the COVID-19 pandemic was considered to be a media hype, this attitude had a negative effect on the vaccination status of the higher education students (aOR = 0.840). In our survey, the most important contribution to the explanation of the COVID-19 vaccination status was having the confidence that the COVID-19 vaccination is safe (aOR = 1.656) and the attitude of making a positive contribution to the course of the pandemic with one’s own vaccination (aOR = 1.650).

**Figure 1:**
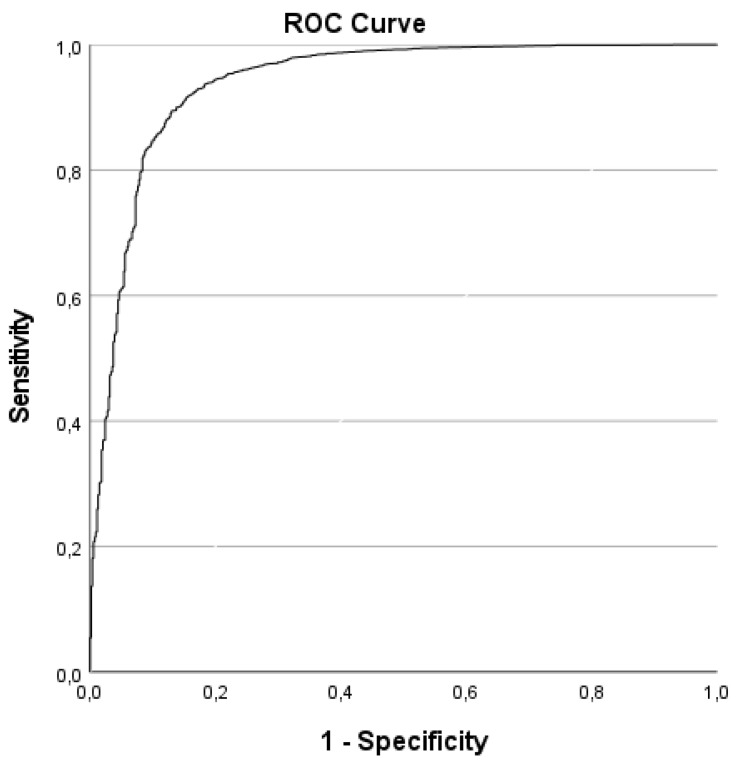
The receiver operating characteristics (ROC) curve with predicted probabilities for the multivariate logistic regression analysis for students’ COVID-19 vaccination status in summer/fall 2021 (n = 6379).

The effect strength of the multivariate binary logistic regression model amounts showed f^2^ = 0.59 and indicates a strong effect on students’ COVID-19 vaccination status. The overall correct classification rate was 95.5%. For the calculation of the receiver operating characteristics (ROC) curve, n = 5841 cases with positive COVID-19 vaccination status and n = 538 cases with negative COVID-19 vaccination status were included; n = 177 cases (2.7%) were excluded that showed a missing value for at least one variable of the model. Figure 1 shows the ROC curve; the AUC measures 0.939. The overall model has a very good model fit with an AUC close to perfect accuracy.

## 4. Discussion

In our online survey of higher education students at six HEIs in Baden-Württemberg (BW) in Germany, attitudes toward the appropriateness of health and safety measures to prevent infections with SARS-CoV-2 were very positive. Overall, the behavioral preventive measures were almost universally rated as excellently suited and the structural preventive measures were rated as well suited. Participating higher education students also indicated high vaccination acceptance in the summer and fall 2021, as a total of 91.4% of respondents had been vaccinated against COVID-19 at least once. These results on behavioral and structural preventive measures indicate broad support of the study participants for a return to face-to-face teaching in HEIs in the winter semester 2021/2022, in compliance with recommended preventive measures related to the COVID-19 pandemic.

Within our study population of higher education students, we examined factors associated with students’ COVID-19 vaccination status in summer/fall 2021. During this time, according to a review published by the Robert Koch Institute—the German federal government agency and research institute for disease control and prevention—the risk of SARS-CoV-2 transmission appeared to be very much reduced by COVID-19 vaccination, suggesting that vaccinated individuals would not play a significant role in the disease’s epidemiology [61,62].

Subsequently, we discuss the development and composition of the final multivariate binary logistic regression model. We first point out variables that were excluded during the selection process within the thematic variable groups (blocks). Secondly, we highlight how perceptions of SARS-CoV-2 in general and attitudes towards health and safety measures implemented to protect higher education students against transmissions of SARS-CoV-2 were associated with their vaccination status.

### 4.1. Variables Not Directly Related to COVID-19

Age was not included in our regression model. Since all our survey participants are higher education students, they have similar prerequisites (at least 12 years of schooling and similar age). We assumed that higher education students are a relatively homogeneous group. In a study among undergraduate students in Italy by Gallè et al., the authors assumed that the age range of their sample was potentially too narrow to observe age differences [15]. A significant effect of age was found only partially in studies of COVID-19 vaccination behavior [22]. A study among Serbian university students [63] found a significant difference in COVID-19 vaccination: older students were more likely to plan vaccination or to have already been vaccinated. For example, in a study analyzing the pandemic’s impact on respondent health behavior, Mercadante et al. [64] found significant differences between age groups and education of respondents on the 5C instrument.

Regarding the relationship between gender and COVID-19 vaccination acceptance, it is usually found that males are more likely to be vaccinated against COVID-19 (e.g., [15,27,65,66]); in some studies, gender no longer contributes significantly to multivariate analyses [12,67]. In our study sample, young women are overrepresented; there are other reasons against COVID-19 vaccination in women than in men (for example pregnancy or the desire to have children) that we did not examine in our survey.

Education levels often indicate that higher education is associated with a positive COVID-19 vaccination status [22].

With regard to the medical or health science study background, it is usually evident that students with healthcare curricula are more likely to be vaccinated against COVID-19; however, studies among dental students indicate rather poor vaccination acceptance [29,68]. Similar to a study among Italian students, we observed no difference in healthcare students versus non-healthcare students in our sample [26].

### 4.2. Perception of SARS-CoV-2 in General and Attitudes toward Health and Safety Measures Implemented to Prevent SARS-CoV-2 Infections in the Study Environment

Affective risk has been shown as a motive for protective behavior and the acceptance of measures [69,70]. This is also evident in our study, where an increased affective risk of COVID-19 contributes to the positive vaccination status of the higher education students. If, on the other hand, the COVID-19 outbreak is seen as media hype, this in turn has the potential to help explain why some people do not get vaccinated against COVID-19.

Although previous literature shows that there are differences in COVID-19 vaccination acceptance and that attitudes toward behavioral and structural preventive measures are important for the acceptance of health and safety measures [71,72,73], COVID-19 vaccination acceptance and behavioral and structural preventive measures against the transmission of SARS-CoV-2 among higher education students have not yet been present in the current literature from Germany. Regarding compliance with preventive measures during the COVID-19 pandemic, a positive correlation was also found between people’s attitudes toward protective and hygienic measures and their adoption of measures to prevent the transmission of SARS-CoV-2 [74,75,76,77]. Results of the COSMO snapshot monitoring of the general population in Germany showed that people who are partially or fully vaccinated against COVID-19 have high protective behavior; for example, rapid antigen testing before large events is more common among vaccinated than unvaccinated individuals [69]. In our study on attitudes toward health and safety measures to prevent SARS-CoV-2 infections in the environment of HEIs, these attitudes contributed positively to the explanation of the COVID-19 vaccination status of our respondents until the vaccination-related variables were added to the model in the last block. A possible explanation for this effect may be related to the widespread belief during the survey period that vaccination reliably protects against infection with SARS-CoV-2 [62]. Furthermore, the strong positive influence of the belief in making an important contribution to pandemic mitigation through the vaccination itself may indicate that the sense of safety in vaccination leads to considering the other preventive protective and hygienic measures to be less important. Together, this leads us to the assumption that the individual variables are predictors of higher education students’ COVID-19 vaccination status, whereas variables on SARS-CoV-2 perception, attitudes toward behavioral and structural preventive measures and COVID-19 vaccination-related variables are more likely to act as mediators. This study could, however, not conclusively clarify how to characterize the impact of these attitudes in interaction with the COVID-19 vaccine-related variables on the vaccination status of the surveyed higher education students.

### 4.3. Relevance

COVID-19 vaccine hesitancy among higher education students may hinder resumption of face-to-face teaching in HEIs. For this reason, it was essential to obtain the student’s attitudes and to communicate them to the universities in a timely manner, so that on-site education and vaccination offers could be adapted and specified to the needs of the students. For example, some HEIs extended their vaccination programs, facilitated access (e.g., vaccination without appointments across various places on campus) or adopted communication strategies [78]. The relevance of concerns about COVID-19 vaccination are also evident in the growing number of publications, and the development and validation of measurement tools [79,80]. Despite relatively high vaccination rates among higher education students, attitudes towards and implementation of behavioral and structural measures to prevent SARS-CoV-2 infections remain highly important. Given that the long-term consequences of the pandemic are not yet clearly assessable (e.g., impact of Long COVID on the current and future workforce), it remains critical to counteract increasing infection rates [81].

Our multivariate binary logistic regression model can predict very well those higher education students who have already received at least one vaccination against COVID-19 (98.5% of accuracy). The students who have not yet received vaccination against COVID-19 can be correctly classified by our regression model only by 62.5%. Reasons for this might be the targeting of the questionnaire on COVID-19 vaccination and the invitations to the survey via the HEIs themselves, which wanted to identify information on the vaccination status of their students.

The presented data from the cross-sectional surveys at the six HEIs provided some insight into the considerations of HEI students at the time when COVID-19 vaccination was offered to all of them. However, this did not occur in the exact same time frame, but over a longer period between July and November 2021.

### 4.4. Limitations

Response bias could be one of the main limitations, as the data are based on voluntary self-reports and were collected online by invitation over the participating HEIs. We assume that students with positive attitudes toward COVID-19 vaccination are overrepresented in our survey. In combination with the varying and relatively low response rates, our results are therefore not generalizable beyond our sample. It is important to emphasize that the vaccination coverage in our study population was very high (91.4%) compared to the general population in Germany; in the federal state of BW, the proportion of individuals in the general population with vaccination recommendation who had received at least one COVID-19 vaccination by 30 August 2021 was 62.5% [82].

It is likely that individuals with negative attitudes towards the pandemic and COVID-19 vaccination would be more likely to decline to participate in the survey. Only a small proportion indicated that they had not yet been vaccinated against COVID-19 and gave detailed reasons in free text why they did not want to be vaccinated at the time of the survey. Generalizability of our findings, of factors influencing COVID-19 vaccination, is not possible because of our specific sample of higher education students in southwestern Germany. Due to the limited time between the development of the questionnaire and the data collections, the survey was conducted in German language only. It is likely that international higher education students are underrepresented in the survey.

The results on COVID-19 vaccination acceptance must always be considered context-dependent and assigned to the respective level of knowledge at the time of the survey. Vaccination acceptance varies widely from country to country and region to region, confirming the definition as “complex and context-specific, varying across time, place and vaccines” [7]. This could be due to the complex and unforeseeable interaction of many demographic, cultural and social factors [24]. Surveys should be repeated and regarded with caution because of the changing and impossible to forecast attitudes towards COVID-19 vaccination [16].

Other factors influencing COVID-19 vaccination acceptance, such as knowledge of COVID-19 vaccination, as found by Gallè et al. [15] among undergraduate students in Italy, were not part of our survey, so we are not able to provide a conclusion on the association with student vaccination behavior for this and other possible predictors.

### 4.5. Strengths

Only a few surveys on vaccination acceptance were conducted among higher education students of all ages, as most focused on specific groups such as undergraduates or students with healthcare curricula (e.g., [10,28,67,83,84]). In Germany, current research on COVID-19 vaccination acceptance is mainly focused on the general population (e.g., [85,86,87]). The factors influencing the vaccination status of higher education students in Germany have not yet been investigated. The strength of our survey is the large sample of higher education students from different fields of study as well as the survey period given that all individuals had been offered vaccination. Compared to other studies focusing on vaccination acceptance among higher education students worldwide, we add to the current knowledge base with data on these attitudes toward behavioral and structural preventive measures in the higher education study environment.

Furthermore, a descriptive analysis of key findings from the online surveys was prepared for each of the participating institutions; so protective measures could be adapted accordingly.

### 4.6. Implications and Future Research

A European Commission report concluded that more research is needed on the impact of learning loss, financial consequences and effects on educational inequities [31]. This study contributes to the understanding of attitudes towards behavioral and structural preventive measures in the context of the COVID-19 pandemic among higher education students in southwestern Germany. The results could be used in education and information campaigns as well as in vaccination campaigns and thus promote both vaccination acceptance and herd immunity of the population in Germany. In the further course of the comprehensive research project [46,47], the attitudes of employees at HEIs toward preventive and hygienic measures during the COVID-19 pandemic will also be related to the attitudes of employees in other companies and workplaces.

## 5. Conclusions

Our study provides new evidence into factors associated with COVID-19 vaccination acceptance. Regardless of field of study or affiliation to higher education institution, the explanatory variables (rather mediators than predictors) for the COVID-19 vaccination status of our study participants were variables on the perception of SARS-CoV-2 in general, attitudes toward behavioral and structural measures to prevent SARS-CoV-2 infections in the study environment and largely attitudes toward COVID-19 vaccination. Our findings contribute to the planning and management of face-to-face teaching over the ongoing course of the COVID-19 pandemic.

## Figures and Tables

**Table 1:** Individual variables—characteristics of higher education students.

Characteristic	Specification	n (%)	Valid Percentage *
Age (years) (n = 6259)	Mean (SD)	24 (4.2)	
	Median	23	
	Range	18–70	
Gender	Female	n = 4106 (62.6)	64.8
	Male	n = 2226 (34.0)	35.2
	Divers	n = 49 (0.7)	
	Missing	n = 175 (2.7)	
Affiliation to risk group for developing severe COVID-19 courses (pre-existing conditions ^1^)	Yes	n = 597 (9.1)	9.4
	No	n = 5764 (87.9)	90.6
	Missing	n = 195 (3.0)	
Confirmed SARS-CoV-2 infection	Yes	n = 312 (4.8)	5.6
	No	n = 5220 (79.6)	94.4
	Missing	n = 1024 (15.6)	
Received at least one dose of COVID-19 vaccine	Yes	n = 5935 (90.5)	91.4
	No	n = 555 (8.5)	8.6
	Missing	n = 66 (1.0)	
Nationality	German	n = 6143 (93.7)	95.2
	Other	n = 308 (4.7)	4.8
	Missing	n = 105 (1.6)	
Part-time job while studying	Yes	n = 2549 (38.9)	39.5
	No	n = 3902 (59.5)	60.5
	Missing	n = 105 (1.6)	
Living in a committed relationship	Yes	n = 3037 (46.3)	48.1
	No	n = 3277 (50.0)	51.9
	Missing	n = 242 (3.7)	
Number of household members	Living alone with no other person	n = 657 (10.0)	10.2
	Living with one other person	n = 1691 (25.8)	26.2
	Living with 3–4 other persons	n = 2854 (43.5)	44.2
	Living with more than 4 other persons	n = 1256 (19.2)	19.4
	Missing	n = 98 (1.5)	
Health professional within household	Yes	n = 1502 (22.9)	23.5
	No	n = 4897 (74.7)	76.5
	Missing	n = 157 (2.4)	
Trait extraversion ^2^ (n = 6495)	Mean (SD)	3.17 (1.04)	
Trait agreeableness ^2^ (n = 6494)	Mean (SD)	3.23 (0.81)	
Trait conscientiousness ^2^ (n = 6494)	Mean (SD)	3.65 (0.83)	
Trait neuroticism ^2^ (n = 6494)	Mean (SD)	3.07 (0.98)	
Trait openness to experiences ^2^ (n = 6490)	Mean (SD)	3.57 (1.02)	

* Valid percentage or valid percentage of the variable used dichotomously (gender: male/female). ^1^ Pre-existing conditions include, for example, specific primary diseases such as cardiovascular disease, diabetes, or diseases of the respiratory system, or suppressed immune systems [44]. ^2^ Big Five personality trait [51]: range 1–5—low to high.

**Table 2:** Model summary for each block of the multivariate binary logistic regression analysis.

Variable Group from the Questionnaire (Step) Block		Hosmer and Lemeshow Test	−2 Log Likelihood	Cox & Snell R Square	Nagelkerke Pseudo R^2^	Area under the ROC Curve (AUC)
Control variables: HEI affiliation with five categorical variables with dummy-coding and two social desirability scores [52]		χ^2^(8) = 2.513 *p* = 0.961	3633.915	0.009	0.020	0.589
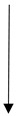	+(II) Perception of SARS-CoV-2 in general (Affective risk perception, Perception of the outbreak as a media-hype)	χ^2^(8) = 48.822 *p* < 0.001	2798.381	0.130	0.297	0.831
	+(III) Attitude toward health and safety measures to prevent SARS-CoV-2 infections (behavioral and structural preventive measures in the study environment)	χ^2^(8) = 48.178 *p* < 0.001	2787.634	0.132	0.300	0.833
	+(V) Variables relating to COVID-19 vaccination (own health, COVID-19 vaccine safety, avoidance of disadvantages of the pandemic for HEIs, contribution to the containment of the pandemic)	χ^2^(8) = 5.424 *p* = 0.711	1698.670	0.268	0.610	0.939

**Table 3:** Multivariate binary logistic regression analysis: possible explanatory variables (factors) and the outcome “COVID-19 vaccination status” (not vaccinated against COVID-19/received at least one dose of COVID-19 vaccine).

Explanatory Variables (Factors) *		Regression-Coefficient B	Wald Statistics	*p*-Value	Adjusted Odds Ratio * (aOR)	95% Confidence Interval for OR	
Variable Group	Variables (Range: Low to High)					Lower Limit	Upper Limit
(II) Perception of SARS-CoV-2 in general ^1^	Affective risk perception	0.161	7.101	0.008	1.175	1.044	1.323
	Perception of the outbreak as a media-hype	−0.174	7.997	0.005	0.840	0.744	0.948
(III) Attitude toward health and safety measures to prevent SARS-CoV-2 infections	Attitude toward behavioral preventive measures in the study environment	−0.310	4.825	0.028	0.733	0.556	0.967
	Attitude toward structural preventive measures in the study environment	−0.247	3.981	0.046	0.781	0.613	0.996
(V) Variables relating to COVID-19 vaccination	Vaccinating against COVID-19 mainly helps to preserve my health. ^1^	0.270	43.230	<0.001	1.310	1.209	1.420
	I am completely confident that vaccination against COVID-19 is safe. ^1,2^	0.505	127.923	<0.001	1.656	1.518	1.808
	Vaccinating against COVID-19 primarily helps higher education to eliminate the disadvantages caused by the pandemic.	0.150	14.582	<0.001	1.161	1.076	1.254
	I contribute to the containment of the pandemic by vaccinating against COVID-19. ^1^	0.501	111.078	<0.001	1.650	1.503	1.811

* The multivariate binary logistic regression model was controlled for affiliation with the six HEIs and response behavior by social desirability [52]. ^1^ COSMO—COVID-19 snapshot monitoring [54]. ^2^ 5C psychological antecedents of vaccination [56] in relation to COVID-19 vaccination.

## Data Availability

Data are not publicly available due to the participants’ informed consent. The data presented in this study are available on reasonable request from the corresponding author.

## Supplementary Materials

The following supporting information can be downloaded at: https://www.mdpi.com/article/10.3390/vaccines10091433/s1, Table S1: Univariate binary logistic regression analysis: possible explanatory variables (factors) and the outcome “COVID-19 vaccination status” (not vaccinated against COVID-19 = 0/received at least one dose of COVID-19 vaccine = 1).
