# Supplementary material for: Factors Associated with the COVID-19 Vaccination Status of Higher Education Students: Results of an Online Cross-Sectional Survey at Six Universities in Southwestern Germany
Source: Vaccines (Basel). 2022 Aug 30;10(9):1433. doi: 10.3390/vaccines10091433 (PMC9505187; doi:10.3390/vaccines10091433)
Supplement: Supplementary file 1 [file vaccines-10-01433-s001.zip › vaccines-1865974-supplementary.pdf]

Supplementary Material

Table S1. Univariate binary logistic regression analysis: possible explanatory variables (factors) and the outcome "COVID-19 vaccination status" (not vaccinated against COVID-19 = 0 / received at least one dose of COVID-19 vaccine = 1)

Explanatory Variables (Factors)		Regression-coefficient B	Wald statistics	*p*-value	Odds Ratio (OR)	95% confidence interval for OR	
Variable Group from the questionnaire	Variables *					Lower limit	Upper limit
(I) Individual variables (socio-demographic, personal and study-related characteristics)	Age	-0.018	3.360	0.067	0.982	0.963	1.001
	Gender (male/female)	-0.035	0.130	0.718	0.966	0.799	1.167
	Affiliation to risk group (0/1)	0.097	0.361	0.548	1.101	0.804	1.509
	German Nationality (0/1)	0.478	7.076	**0.008**	1.613	1.134	2.293
	Employment (0/1)	0.221	5.529	**0.019**	1.247	1.037	1.499
	Health professionals within household (0/1)	-0.217	4.500	**0.034**	0.805	0.659	0.984
	Relationship (0/1)	-0.284	9.566	**0.002**	0.753	0.629	0.901
	Trait extraversion [1]	-0.085	3.877	**0.049**	0.919	0.845	1.000
	Trait agreeableness [1]	0.025	0.201	0.654	1.025	0.921	1.141
	Trait conscientiousness [1]	-0.415	57.711	**<0.001**	0.661	0.591	0.739
	Trait neuroticism [1]	0.253	30.127	**<0.001**	1.287	1.176	1.409
	Trait openness to experiences [1]	-0.100	4.943	**0.026**	0.905	0.829	0.988
	Higher education institution (application-oriented / research-oriented)	0.001	0.000	0.991	1.001	0.776	1.292
	Healthcare university curriculum (0/1)	0.579	10.676	**0.001**	1.785	1.261	2.527
(II) Perception of SARS-CoV-2 in general [2]	Disease perception	0.738	384.151	**<0.001**	2.091	1.942	2.251
	Affective risk perception	0.815	479.489	**<0.001**	2.258	2.099	2.429
	Perception of the outbreak as a media-hype [†]	-1.033	564.087	**<0.001**	0.356	0.327	0.388
	Perceived personal susceptibility to contract SARS-CoV-2 [†]	0.247	54.104	**<0.001**	1.280	1.199	1.368
	Expected severity of the disease [†]	0.553	202.391	**<0.001**	1.738	1.611	1.876
(III) Attitude toward health and safety measures to prevent SARS-CoV-2 infections	Attitude toward behavioral preventive measures in the study environment	1.050	240.600	**<0.001**	2.858	2.503	3.264
	Attitude toward structural preventive measures in the study environment	0.975	263.585	**<0.001**	2.643	2.349	2.975
(IV) Impact of COVID-19 on the personal environment	Perceived probability to contract COVID-19 in private surrounding [†]	0.247	62.003	**<0.001**	1.281	1.204	1.362
	Perceived probability to contract COVID-19 in study environment [†]	0.031	1.259	0.262	1.031	0.977	1.088
	Readiness to perform SARS-CoV-2 rapid antigen tests [†]	0.397	336.604	**<0.001**	1.488	1.426	1.552
	Reactance regarding measures taken to prevent SARS Cov-2 infections	-0.641	420.110	**<0.001**	0.527	0.496	0.560
	COVID-specific resilience	-0.059	2.835	0.092	0.942	0.880	1.010
	Positive assessment of fellow students to adhere to the distance rules in the course of studies [†]	-0.123	21.749	**<0.001**	0.885	0.840	0.931

	Positive assessment of fellow students to adhere to the hygiene rules in the course of studies †	-0.003	0.014	0.905	0.997	0.942	1.054
(V) Variables relating to COVID-19 vaccination	Vaccinating against COVID-19 mainly helps to preserve my health. † 2	0.803	872.118	**<0.001**	2.233	2.117	2.355
	Vaccinating against COVID-19 mainly helps to eliminate the disadvantages I have due to the pandemic. † 2	0.419	327.183	**<0.001**	1.520	1.453	1.591
	Vaccinating against COVID-19 primarily helps higher education institutions to eliminate the disadvantages caused by the pandemic. †	0.652	664.437	**<0.001**	1.920	1.827	2.018
	I am completely confident that vaccination against COVID-19 is safe. † 2,3	1.007	1019.599	**<0.001**	2.738	2.574	2.913
	Vaccination against COVID-19 is unnecessary because COVID-19 is not a major threat. † 2,3	-0.892	911.358	**<0.001**	0.410	0.387	0.434
	Everyday stress prevents me from getting vaccinated against COVID-19. † 2,3	-0.521	297.297	**<0.001**	0.594	0.560	0.630
	When I think about getting vaccinated against COVID-19, I weigh benefits and risks to make the best decision possible. † 2,3	-0.632	258.614	**<0.001**	0.531	0.492	0.574
	When everyone is vaccinated against COVID-19, I don't have to get vaccinated, too. † 2,3	-0.729	748.002	**<0.001**	0.482	0.458	0.508
	Vaccination allows us to return to routine life. † 2	0.846	932.675	**<0.001**	2.331	2.208	2.461
	Due to the COVID-19 vaccination, I can have more social contacts again. † 2	0.865	963.919	**<0.001**	2.376	2.249	2.509
	I contribute to the containment of the pandemic by vaccinating against COVID-19. † 2	0.952	1103.677	**<0.001**	2.590	2.448	2.739

* 0 = no; 1 = yes

† Measurement on 7-point Likert scale with range low to high

1 Big-Five-Trait [51]: range 1-5 – low to high

2 COSMO — COVID-19 Snapshot Monitoring [54]

3 5C psychological antecedents of vaccination [56] in relation to COVID-19 vaccination
